# Dietary pattern and lifestyle risk factors for sessile serrated precursors to colorectal cancer

**DOI:** 10.1017/S0007114525103747

**Published:** 2025-07-14

**Authors:** Jolieke C. van der Pols, Elizabeth A. Johnston, Vicki L. J. Whitehall, Torukiri I. Ibiebele, David G. Hewett, Barbara A. Leggett

**Affiliations:** 1 School of Exercise and Nutrition Sciences, Queensland University of Technology, Brisbane, QLD, Australia; 2 Population Health Program, QIMR Berghofer Medical Research Institute, Herston, QLD, Australia; 3 Viertel Cancer Research Centre, Cancer Council Queensland, Fortitude Valley, QLD, Australia; 4 Conjoint Gastroenterology Laboratory, QIMR Berghofer Medical Research Institute, Herston, QLD, Australia; 5 School of Medicine, The University of Queensland, Herston, QLD, Australia; 6 Department of Gastroenterology, Royal Brisbane and Women’s Hospital, Herston, QLD, Australia

**Keywords:** Bowel cancer, Cancer risk, Colorectal polyps, Epidemiology, Sessile serrated adenoma

## Abstract

Sessile serrated lesions (SSL) are recognised precursors to colorectal cancer. Little is known about risk factors for SSL due to their relatively recent clinical recognition as a cancer precursor and routine documentation of cases. Lifestyle and diet-related information were collected using validated questionnaires in a colonoscopy-based case–control study in Australia (257 SSL cases, 239 conventional adenoma (CA) cases, 180 polyp-free controls). *A posteriori* dietary patterns were derived from self-reported dietary intake in the past 12 months using principal component analysis. Multivariable-adjusted OR and 95 % CI were used to examine associations between lifestyle factors and dietary patterns on risk of SSL and CA *v*. polyp-free controls and SSL *v*. CA using logistic regression modelling. Use of anti-inflammatory medications was associated with reduced SSL risk (OR = 0·61; 95 % CI 0·38, 1·00), while current smoking was associated with increased SSL risk (OR = 1·96; 95 % CI 1·09, 3·53). Unlike CA, SSL risk was not increased by hormone replacement therapy use and current alcohol consumption but was increased by taller height. Higher adherence to a dietary pattern featuring processed meats, ready-made convenience foods and high-energy drinks was associated with increased SSL risk (OR = 2·13; 95 % CI 1·13, 4·00; *P*_trend_ = 0·03) and CA (OR = 2·60; 95 % CI 1·32, 5·09; *P*_trend_ = 0·005). Compared with CA, a dietary pattern featuring wholegrains, low-fat dairy products, nuts, seeds and oily fish was associated with reduced SSL risk (OR = 0·60; 95 % CI 0·36, 0·98; *P*_trend_ = 0·04). This study supports a healthy diet as primary prevention for both SSL and CA and reinforces smoking as a risk factor for SSL.

Colorectal cancer is a leading cause of cancer-related morbidity and mortality in most Western countries^(1)^. It develops from precursor polyps via multiple pathways. In the most common and long-established pathway, carcinoma develops from tubular adenomas (commonly referred to as conventional adenoma (CA)), accounting for most sporadic (non-hereditary) colorectal cancers^(2)^. Colorectal cancers can also arise via an alternative ‘serrated pathway’, which predominantly includes sessile serrated lesions (SSL), a type of polyp that is typically flat and serrated in appearance. Recent evidence indicates that SSL are responsible for 15–25 % of all sporadic colorectal cancers^(2–4)^.

In many countries, high-risk groups for colorectal cancer are advised to undergo regular colonoscopies for detection and removal of precancerous lesions, while low-risk groups are encouraged to complete a faecal occult blood test (FOBT), with colonoscopy recommended for those returning a positive FOBT test. However, the efficacy of this approach is limited by low participation rates in national FOBT screening programmes^(5)^ and by the very flat morphology of SSL, which makes it more likely for them to be missed during endoscopic examination, increasing the risk of colorectal cancers arising after colonoscopy^(6,7)^. In addition, SSL do not typically lead to positive FOBT, unlike advanced CA. An alternative approach for the prevention of colorectal cancer precursors is modification of diet and lifestyle factors^(8)^. SSL have unique molecular features^(9)^; however, research of epidemiological risk factors has been limited to date due to their relatively recent recognition as colorectal cancer precursors^(10)^. Thus, many past studies of colorectal polyps have not distinguished SSL from other polyp types.

Current evidence of lifestyle and diet-related risk factors of SSL is based on a small number of studies with limited numbers and often poorly defined SSL cases. Thus far, evidence suggests that smoking may increase risk, while regular use of non-steroidal anti-inflammatory drugs may afford protection^(8,11–13)^. Intake of red meat (especially when consumed well-done) and processed meats may increase the risk of SSL^(11,14)^. However, the study of single foods in relation to cancer risk is limited as intake of different foods is often highly correlated, which may confound effect estimates^(15)^. Given the characteristic methylation patterns of SSL and the relevance of folate to methylation patterns in cancer types^(16,17)^, folate and vitamin B_12_ status may also be of relevance.

Dietary pattern analysis is an alternative approach that considers the complex, synergistic interactions between dietary components^(18)^. Previous research has investigated dietary patterns in relation to the risk of any type of colorectal adenoma. In a meta-analysis of seven observational studies for *a posteriori* dietary patterns, unhealthy dietary patterns, characterised by red and processed meats and refined grains, were associated with a significantly higher risk of colorectal adenoma^(19)^. In contrast, healthy dietary patterns, featuring fruit and non-starchy vegetables, were associated with a significantly lower risk of any colorectal adenoma^(19)^. To date, dietary patterns have not been studied in relation to SSL specifically. This study uses data from a case–control study to identify lifestyle-related risk factors and *a posteriori* dietary patterns associated with the risk of SSL in Australian adults.

## Material and methods

### Study design and participants

We recruited study participants aged 18–85 years from patients who had a colonoscopy at one of three major hospitals in Queensland, Australia, between November 2012 and February 2015. The participants in this study had been referred for colonoscopy at these public hospitals for a wide variety of clinical reasons that were not recorded electronically but represented a usual mix of reasons for referral, with many participants having a personal or family history of bowel polyps (Table 1). Recruitment took place prospectively by monthly review of all colonoscopy reports to identify eligible persons. All eligible SSL cases were invited to participate in the study. A matching number of cases (1:1) with CA and an equal number of controls with no polyps were also selected at random from these lists. An invitation letter, information sheet and consent forms were sent to eligible persons. A research nurse subsequently contacted the participants to discuss the study. A self-administered, structured lifestyle questionnaire and FFQ were sent via mail to persons who returned a signed consent form.

**Table 1.** Descriptive characteristics of the study participants (*n* 724)^*^ (Numbers and percentages; mean values and standard deviations)

	Polyp-free controls (*n* 192)		Conventional adenoma (CA) cases (*n* 261)		Sessile serrated lesion (SSL) cases (*n* 271)		
	Mean	sd	Mean	sd	Mean	sd	*P* ^†^
Age (years), mean (sd)	57	13	63	10	59	14	< 0·001
	*n*	%	*n*	%	*n*	%	
Sex (% females)	113	59 %	105	40 %	152	56 %	< 0·001
Caucasian/white ethnicity	171	89 %	236	91 %	248	92 %	0·60
Education							
High school or less	98	52 %	156	60 %	156	58 %	0·26
Vocational training/TAFE	39	21 %	51	20 %	58	21 %	
University degree	53	28 %	51	20 %	57	21 %	
Personal history of polyps	80	42 %	158	61 %	181	68 %	< 0·001
Family history of bowel cancer	70	40 %	63	27 %	100	40 %	0·005
Personal history of cancer	49	26 %	74	28 %	80	30 %	0·66
Prior colonoscopy	152	80 %	176	68 %	210	77 %	0·005
Prior removed part colon/rectum	6	3 %	5	2 %	9	3 %	0·58
Prior removed gallbladder	32	17 %	33	13 %	36	13 %	0·42
History of high cholesterol	75	39 %	136	53 %	104	40 %	0·002
History of diabetes	22	11 %	42	16 %	35	13 %	0·33
	Mean	sd	Mean	sd	Mean	sd	
Energy intake (MJ/d)^‡^	11·0	3·0	10·8	3·1	11·0	3·2	0·72

TAFE, technical and further education; MJ, megajoules; pmol, picomole; nmol, nanomole.

* Values are numbers (%) unless otherwise indicated.

† *P*-values from *χ* ^2^ test for categorical variables, from ANOVA for continuous variables.

‡ Mean (sd) for persons who returned a valid FFQ.

Exclusion criteria included unsatisfactory bowel preparation prior to colonoscopy, inflammatory bowel disease (any type), coeliac disease, a personal history of colorectal cancer, diagnosis of any cancer in the past 12 months (except keratinocyte skin cancers) and family history of familial adenomatous polyposis or Lynch syndrome. Participants were excluded from analyses if the time between colonoscopy and questionnaire completion was more than 52 weeks. This study was conducted according to the guidelines laid down in the Declaration of Helsinki, and all procedures involving human participants were approved by the Human Research Ethics Committees of the QIMR Berghofer Medical Research Institute (P1463), associated universities and the three hospital sites. Written informed consent was obtained from all participants.

### Case definitions

Clinical histology reports were extracted from medical records. If a participant had at least one of the following types of polyps and no serrated lesions, they were classified as an adenoma case: tubular adenoma, tubulovillous adenoma or villous adenoma. SSL cases were defined as a person with at least one lesion that displayed exaggerated crypt serration, crypt dilatation, crypt branching, horizontal crypt extensions at the base or other distortions of architectural organisation and maturation that rendered them distinct from other serrated lesions^(20)^. Polyp-free controls were persons in whom colonoscopy was successfully completed to the caecum, and no polyps were identified.

### Lifestyle data

Participants were asked to complete a self-administered, structured questionnaire that collected information on general personal characteristics (age, sex, ethnicity, education); personal and family history of polyps, bowel cancer and any cancer; prior colonoscopy or removal of the colon, rectum or gallbladder; personal and family history of relevant medical conditions; drug and multivitamin use; and lifestyle factors. The questionnaire was sent by post and returned to the investigators in a reply-paid envelope. Specifically, the questionnaire asked about regular use (i.e. ever taken at least twice per week for a month or longer) of aspirin, anti-inflammatory pain killers, multivitamins and hormone replacement therapy (HRT). Current alcohol consumption was assessed as consuming beer, wine, spirits, shots, cocktails or mixed drinks (yes/no). Smoking duration and frequency were summarised as never, former (smoked one or more cigarettes a day for 3 months or longer, but not a current smoker) or current smoker (currently smokes at least one cigarette a day).

Current height and weight were self-reported and used to calculate BMI (kg/m^2^). BMI was categorised using WHO recommendations (normal, overweight, obese)^(21)^. Three tertile groups for height (short, average, tall) were created based on ranked self-reported height in centimetres stratified by sex. Physical activity was assessed as metabolic equivalent of task (MET) values (i.e. the ratio of work metabolic rate to a standard RMR of 1·0 kcal/kg/h) according to the Compendium of Physical Activities^(22)^. MET-hours per week for each participant were generated using participants’ reports of the frequency and duration (in hours and minutes) of three categories of exercise (walking, moderate and vigorous activities) during the past week. Study participants were stratified by sex and then ranked into approximately equal thirds based on their MET-hours per week to derive physical activity categories (low, medium, high). A blood sample was collected in serum and EDTA tubes at a participant’s local pathology clinic. This was transported to the Royal Brisbane and Women’s Hospital Central Pathology Laboratory for analysis of red blood cell folate using the Beckman Coulter Folate Plasma and Serum Enzyme Immunoassay.

### Dietary data

The usual diet in the past 12 months was assessed using a self-completed, 129-item semi-quantitative FFQ. The FFQ has been validated against weighed food records for use in cancer population studies in Australia^(23)^ and used previously in our cancer studies^(24,25)^. The FFQ asked participants to nominate a frequency of consumption for each food item from ‘never’ to ‘4+ per day’ based on a standard serving size. Brand and type of any butter, margarine or breakfast cereals were collected and matched with Australian Food Composition Database codes^(26)^. Four additional questions included the quantity of sugar added to food or beverages and the frequency of consumption of visible fat on meat, fried foods at home and take-away fried foods. Two further questions assessed the frequency of red meat intake in a typical week (‘never’ to ‘6 or more times’) and preferences for cooked meat (pink or lightly browned, medium browned, deep brown or blackened).

### Assessment of dietary patterns

Food frequency data were converted to daily intake and then multiplied by the standard portion size specified in the FFQ to obtain daily intake in grams. Seasonal fruits were weighted according to the proportion of the year the fruit is available in Australia^(27)^. Total daily energy intake was calculated using Australia’s reference nutrient database, NUTTAB 2010^(28)^. Nutrient intakes were adjusted for energy intake using the residual method. Using the same criteria as in our previous studies^(29,30)^, participants with implausible calculated energy intake (< 3360 kJ/d or > 21 000 kJ/d for men, < 2940 kJ/d or > 16 800 for women) were excluded.

The 129 food items and 4 additional sugar and fat items were reclassified into 34 pre-defined food groups based on nutrient composition and culinary use (online Supplementary Table 1). Foods and beverages with distinct nutrient composition (e.g. tea, coffee, Vegemite) were retained as individual items. The average daily intake of each food group for each participant was calculated by summing the intake of individual foods in each group. Food group intakes were log transformed to improve normality. Principal component analysis was conducted on the thirty-four food groups using PROC FACTOR and METHOD = PRINCIPAL in SAS University Edition (SAS Institute). Criteria for retaining factors (dietary patterns) were derived from previous studies and included eigenvalue > 1·75, break point of scree plot, proportion of variance explained and factor interpretability. Retained factors underwent varimax rotation to obtain an orthogonal solution. Factors were labelled based on food groups with absolute factor loadings of 0·30 or greater, as recommended by Garcia-Larsen and colleagues^(31)^. A factor score was calculated for each participant by summing the intake of each food group weighted by its factor loading, with higher factor scores indicating higher adherence to the dietary pattern. Factor scores were separated into tertiles for statistical analysis, with the lowest tertile used as the reference category.

### Statistical analysis

The sample size for this study was informed by similar studies previously published^(11,32)^ and power calculations that indicated with an estimated 250 cases and 250 polyp-free control persons, expected exposure among controls on average 20 %, power of 80 % and two-sided significance of 0·05, the study would have detectable OR of 0·49 and smaller or 1·79 and larger (PS Power and Sample Size software, Vanderbilt University). Descriptive statistics were used to report the general characteristics of participants in the three study groups (CA, SSL, polyp-free control). *χ* ^2^ test was used to assess differences in the general characteristics of the study participants between the three study groups. ANOVA and Kruskal–Wallis tests were performed to identify group differences for continuous variables that either had a normal or non-normal distribution, respectively.

Binary and multinomial logistic regression models were used to obtain OR and 95 % CI to assess associations between lifestyle factors and the risk of SSL and CA compared with polyp-free controls (case–control comparisons) and risk of SSL compared with CA (case-case comparison). The multivariable model adjusted for age (years), sex (male/female), personal polyp history (yes/no), family history of bowel cancer (yes/no), previous colonoscopy (yes/no), smoking status (never/former/current), current alcohol consumer (yes/no), BMI categories (< 25/25–39/≥ 30), use of anti-inflammatory medication (yes/no) and regular aspirin use (yes/no). These covariates were identified from factors reported in the literature to influence adenoma risk, as well as empirically tested confounders within the dataset.

Multinomial logistic regression was used to obtain OR and 95 % CI to assess associations between tertiles of dietary pattern scores and risk of SSL and CA using the same case–control comparisons and case-case comparison. The first model was adjusted for age and sex only. The multivariable model adjusted for age (years), sex (male/female), total energy intake (megajoules/d), BMI category (< 25/25–39/≥ 30), smoking status (never/former/current), personal polyp history (yes/no), bowel cancer history (yes/no), previous colonoscopy (yes/no), current alcohol consumer (yes/no) and regular aspirin use (yes/no). These covariates were identified from factors reported in the literature to influence adenoma risk, as well as empirically tested confounders within the dataset. Continuous covariates were centred on the mean. Linear trends were assessed by assigning the numbers 1–3 for the lowest to highest tertile groups, respectively, and modelling this as a continuous variable. Analyses were carried out using the SAS statistical package version 9·4 (SAS Institute). Two-tailed *P* ≤ 0·05 indicated statistical significance for all tests.

## Results

A total of 1813 persons were invited to participate in this study, including 598 SSL cases, 599 CA cases and 616 polyp-free controls. A total of 735 persons consented to participate in the study, from which 10 persons with a personal history of colorectal cancer and one person who took > 52 weeks to return their questionnaires were excluded. Therefore, 724 persons (40 %) were included in the current analyses of lifestyle factors, including 271 SSL cases (45 %), 261 CA cases (44 %) and 192 polyp-free controls (31 %). Compared with CA cases, SSL cases were on average younger, more likely to be female, more likely to have had a personal history of polyps or a family history of bowel cancer, more likely to have had a prior colonoscopy and less likely to have a history of high cholesterol levels (Table 1).

Compared with polyp-free controls, regular use of anti-inflammatory medication was associated with reduced risk of SSL (OR = 0·61; 95 % CI 0·38, 1·00), while being a current smoker was associated with increased risk of SSL (OR = 1·96; 95 % CI 1·09, 3·53) (Table 2). Compared with CA cases, a history of regular use of anti-inflammatory medications (OR = 0·63; 95 % CI 0·40, 1·00), current alcohol consumption (OR = 0·63; 95 % CI 0·42, 0·97) and being overweight (OR = 0·55; 95 % CI 0·35, 0·88) were associated with reduced risk of SSL. Being a current smoker (OR = 1·89; 95 % CI 1·09, 3·29) and being taller (OR = 1·63; 95 % CI 1·04, 2·57) were associated with increased risk of SSL (Table 2).

**Table 2.** Associations between medication use, lifestyle factors and risk of adenoma types (*n* 724)^*^ (Numbers and percentages; odds ratios and 95 % confidence intervals)

	Polyp-free controls (*n* 192)		CA cases (*n* 261)		SSL cases (*n* 271)			CA *v*. controls Adjusted OR^*^		SSL *v*. controls Adjusted OR^*^		SSL *v*. CA cases Adjusted OR^*^	
	*n*	%	*n*	%	*n*	%	*P* ^†^	OR	95 % CI	OR	95 % CI	OR	95 % CI
Regular use of aspirin													
No	145	77 %	149	64 %	171	70 %	0·02	1·00 (ref.)		1·00 (ref.)		1·00 (ref.)	
Yes	44	23 %	83	36 %	74	30 %		1·16	0·67, 1·86	0·97	0·59, 1·62	0·88	0·60, 1·36
Regular use of anti-inflammatory medications													
No	131	70 %	165	71 %	186	78 %	0·005	1·00 (ref.)		1·00 (ref.)		1·00 (ref.)	
Yes	55	30 %	67	29 %	54	23 %		0·98	0·60, 1·60	0·61	0·38, 1·00	0·63	0·40, 1·00
Use of multivitamin supplements													
No	124	65 %	160	68 %	164	66 %	0·86	1·00 (ref.)		1·00 (ref.)		1·00 (ref.)	
Yes	66	35 %	76	32 %	83	34 %		0·98	0·61, 1·56	1·00	0·64, 1·57	1·05	0·69, 1·61
Use of HRT													
No	86	79 %	63	70 %	99	78 %	0·28	1·00 (ref.)		1·00 (ref.)		1·00 (ref.)	
Yes	23	21 %	27	30 %	28	22 %		1·05	0·49, 2·25	0·94	0·46, 1·93	0·63	0·32, 1·25
Current alcohol consumption													
No	71	37 %	69	27 %	91	34 %	0·03	1·00 (ref.)		1·00 (ref.)		1·00 (ref.)	
Yes	119	63 %	188	73 %	170	65 %		1·85	1·15, 3·00	1·17	0·76, 1·80	0·63	0·42, 0·97
Smoking													
Never smoker	100	52 %	105	40 %	107	40 %	0·01	1·00 (ref.)		1·00 (ref.)		1·00 (ref.)	
Former smoker	64	33 %	122	47 %	107	40 %		1·38	0·87, 2·20	1·47	0·92, 2·34	1·04	0·69, 1·56
Current smoker	28	15 %	33	13 %	55	20 %		1·09	0·56, 2·16	1·96	1·09, 3·53	1·89	1·09, 3·29
Weight status (BMI)													
Normal weight (BMI < 25)	67	37 %	68	28 %	96	37 %	0·02	1·00 (ref.)		1·00 (ref.)		1·00 (ref.)	
Overweight (BMI 25–29)	66	36 %	101	41 %	71	27 %		1·29	0·76, 2·19	0·80	0·49, 1·32	0·55	0·35, 0·88
Obese (BMI ≥ 30)	48	27 %	78	32 %	93	36 %		1·48	0·84, 2·59	1·45	0·87, 2·42	0·88	0·56, 1·39
Height, cm													
Short (F ≤ 160, M ≤ 173)	63	34 %	105	42 %	87	33 %	0·21	1·00 (ref.)		1·00 (ref.)		1·00 (ref.)	
Average (F ≤ 165, M ≤ 179)	64	35 %	73	29 %	74	28 %		0·79	0·46, 1·35	1·01	0·60, 1·69	1·11	0·70, 1·78
Tall (F > 165, M > 179)	58	31 %	73	29 %	101	39 %		0·81	0·47, 1·41	1·43	0·85, 2·39	1·63	1·04, 2·57
Physical activity (MET h/week)													
Low (F ≤ 13·5, M ≤ 11·5)	62	33 %	88	34 %	89	33 %	0·70	1·00 (ref.)		1·00 (ref.)		1·00 (ref.)	
Medium (F ≤ 35·0, M ≤ 38·5)	57	30 %	90	35 %	94	35 %		1·39	0·82, 2·37	1·21	0·72, 2·02	1·02	0·65, 1·60
High (F ≥ 35·0, M ≥ 39·0)	71	37 %	82	32 %	88	32 %		0·66	0·39, 1·13	0·95	0·58, 1·56	1·15	0·73, 1·83

CA, conventional adenoma; SSL, sessile serrated lesion; F, female; M, male.

* Frequency counts and percentages excluding missing data with adjusted OR and 95 % CI from logistic regression models adjusting for age, sex, personal polyp history, family history of bowel cancer, previous colonoscopy, smoking status, current alcohol consumption, BMI category, use of anti-inflammatory medication and regular aspirin use.

† *P*-values from *χ* ^2^ test for categorical variables.

Current alcohol consumption was a risk factor for CA when compared with polyp-free controls (OR = 1·85; 95 % CI 1·15, 3·00), but none of the other risk factors were associated with CA risk. Regular use of aspirin, use of multivitamin supplements and physical activity level were not associated with the risk of CA or SSL (Table 2).

There were no differences between cases and polyp-free controls for red-cell folate status after adjusting for age, sex, smoking status, alcohol consumption, multivitamin use and the interaction effect between case–control status and multivitamin use (adjusted mean for SSL cases 1475 nmol/l (95 % CI 1395, 1556 nmol/l), CA cases 1435 nmol/l (95 % CI 1355, 1515 nmol/l), polyp-free controls 1438 nmol/l (95 % CI 1340, 1535 nmol/l), *P* > 0·05). Similarly, there were no differences between cases and polyp-free controls for serum vitamin B_12_ status (adjusted mean for SSL cases 263 pmol/l (95 % CI 276, 281 pmol/l), CA cases 270 pmol/l (95 % CI 251, 288 pmol/l), polyp-free controls 275 pmol/l (95 % CI 251, 300 pmol/l), *P* > 0·05) (results not shown in table).

Compared with CA cases, the second tertile of poultry intake (16–44 g/d) was associated with a lower risk of SSL (OR = 0·60; 95 % CI 0·38, 0·95). The highest tertile of oily fish intake (≥ 20 g/d) was associated with a lower risk of SSL, but this was not statistically significant (OR = 0·68; 95 % CI 0·42, 1·09). Frequency of red meat consumption and cooking preference for red meat were not associated with the risk of SSL or CA (Table 3).

**Table 3.** Associations between meat, poultry, oily fish consumption and risk of adenoma types (*n* 676)^*^ (Numbers and percentages; odds ratios and 95 % confidence intervals)

	Polyp-free controls *n* 180		CA cases *n* 239		SSL cases *n* 257			CA *v*. controls		SSL *v*. controls		SSL *v*. CA cases	
	*n*	%	*n*	%	*n*	%	*P*-value^†^	OR^‡^	95 % CI	OR^‡^	95 % CI	OR^‡^	95 % CI
Meat intake amount, g/d (min, max)													
Total meat													
T1: (0, < 122)	63	35 %	84	35 %	78	30 %	0·68	1·00		1·00		1·00	
T2: (122, < 184)	55	31 %	78	33 %	93	36 %		0·83	0·48, 1·46	1·17	0·69, 1·98	1·45	0·90, 2·33
T3: (≥ 184)	62	34 %	77	32 %	86	33 %		0·81	0·45, 1·46	1·03	0·60, 1·78	1·34	0·80, 2·23
Red meat													
T1: (0, < 65)	67	37 %	84	35 %	75	29 %	0·45	1·00		1·00		1·00	
T2: (65, < 113)	58	32 %	77	32 %	90	35 %		0·81	0·47, 1·42	1·26	0·74, 2·14	1·48	0·92, 2·39
T3: (≥ 113)	55	31 %	78	33 %	92	36 %		1·02	0·57, 1·83	1·44	0·85, 2·44	1·50	0·92, 2·46
Processed meat													
T1: (0, < 12)	59	33 %	80	33 %	86	33 %	0·99	1·00		1·00		1·00	
T2: (12, < 29)	61	34 %	82	34 %	85	33 %		0·88	0·51, 1·54	1·00	0·59, 1·70	1·19	0·74, 1·91
T3: (≥ 29)	60	33 %	77	32 %	86	33 %		0·85	0·48, 1·51	0·86	0·50, 1·49	1·28	0·79, 2·09
Poultry													
T1: (0, < 16)	54	30 %	73	31 %	90	35 %	0·50	1·00		1·00		1·00	
T2: (16, < 45)	71	39 %	96	40 %	85	33 %		1·41	0·82, 2·44	0·74	0·45, 1·23	0·60	0·38, 0·95
T3: (≥ 45)	55	31 %	70	29 %	82	32 %		1·32	0·72, 2·43	0·99	0·56, 1·75	0·74	0·44, 1·24
Oily fish													
T1: (0, < 9)	55	31 %	84	35 %	96	37 %	0·61	1·00		1·00		1·00	
T2: (9, < 20)	59	33 %	72	30 %	81	32 %		0·81	0·47, 1·41	0·81	0·48, 1·35	0·86	0·54, 1·37
T3: (≥ 20)	66	37 %	83	35 %	80	31 %		0·95	0·55, 1·64	0·83	0·49, 1·40	0·68	0·42, 1·09
Red meat consumption (number of times per week)													
2 or less times	81	46 %	103	44 %	94	37 %	0·14	1·00		1·00		1·00	
3–5 times	89	50 %	113	48 %	146	57 %		0·81	0·50, 1·30	1·48	0·95, 2·30	1·56	1·03, 2·35
6 or more times	8	4 %	20	8 %	17	7 %		1·39	0·52, 3·67	1·65	0·63, 4·30	0·94	0·44, 2·01
Cooking preference for red meat^‡^													
Pink/lightly browned	20	11 %	28	12 %	24	9 %	0·71	1·00		1·00		1·00	
Medium browned	116	64 %	153	64 %	172	67 %		0·93	0·45, 1·93	1·16	0·57, 2·36	1·32	0·70, 2·48
Deep brown/blackened	33	18 %	47	20 %	53	21 %		1·09	0·47, 2·54	1·37	0·61, 3·09	1·30	0·63, 2·69

CA, conventional adenoma; SSL, sessile serrated lesion; g, grams. min, minimum; max, maximum; T1, tertile 1; T2, tertile 2; T3, tertile 3.

* Frequency counts and percentages with adjusted OR and 95 % CI from logistic regression models adjusting for age, sex, personal polyp history, family history of bowel cancer, previous colonoscopy, smoking status, current alcohol consumption, BMI category, use of anti-inflammatory medication and regular aspirin use.

† *P*-value from *χ* ^2^ test.

‡ In persons with a valid FFQ.

### Dietary pattern analyses

Of the 724 participants, 676 were included in the dietary pattern analyses. Forty-seven participants were excluded because they had an implausible energy intake level, and one person did not complete the FFQ, leaving 257 SSL cases, 239 CA cases and 180 polyp-free controls in these analyses.

Three major dietary patterns were identified: ‘Vegetables and protein sources’, ‘Grains and dairy products’ and ‘Processed meat and discretionary foods’. Online Supplementary Table 2 presents the factor loading matrix for the food groups in each pattern. The ‘Vegetables and protein sources’ pattern was characterised by vegetables, red meat, poultry and seafood. The ‘Grains and dairy products’ pattern featured wholegrains, low-fat dairy products, nuts, seeds and oily fish. The ‘Processed meat and discretionary foods’ pattern included processed meats, ready-made convenience foods, high-energy drinks and fat spreads. These patterns explained 28 % of the total variance in dietary intake among the study population (10·4 % for the first pattern, 9·0 % for the second pattern and 8·7 % for the third pattern). A fourth pattern did not materially increase the total proportion of variance explained by the model.

The ‘Processed meat and discretionary foods’ pattern was associated with more than 2-fold increase in the odds of developing both SSL and CA, with a stronger association identified for CA (OR = 2·13, 95 % CI 1·13, 4·00 for SSL; OR = 2·60, 95 % CI 1·32, 5·09 for CA, highest compared with lowest tertile) (Table 4). When SSL cases were compared with CA cases, the difference in effect estimates for higher adherence to the ‘Processed meat and discretionary food’ pattern was not significant (*P*_trend_ = 0·96). In case-case comparisons, higher adherence to the ‘Grains and dairy products’ pattern was associated with a reduced risk of SSL compared with CA (OR = 0·60; 95 % CI 0·36, 0·98). No associations were identified with higher adherence to the ‘Vegetables and protein sources’ and the ‘Grains and dairy products’ patterns when comparing adenoma groups to polyp-free controls.

**Table 4.** Adjusted OR and 95 % CI for the associations between dietary patterns and adenoma risk

	Case–control comparisons											Case–case comparisons			
	Polyp-free controls (*n* 180)	Conventional adenomas (CA) (*n* 239)					Sessile serrated lesions (SSL) (*n* 257)					SSL *v* CA			
Dietary pattern	*n*	*n*	OR^*^	95 % CI	OR^†^	95 % CI	*n*	OR^*^	95 % CI	OR^†^	95 % CI	OR^*^	95 % CI	OR^†^	95 % CI
Vegetables and protein sources															
T1	61	80	1·00 (ref)		1·00 (ref)		84	1·00 (ref)		1·00 (ref)		1·00 (ref)		1·00 (ref)	
T2	62	84	1·08	0·66, 1·78	1·18	0·68, 2·06	80	0·96	0·60, 1·53	0·98	0·58, 1·64	0·85	0·54, 1·32	0·81	0·50, 1·30
T3	57	75	1·07	0·64, 1·79	1·14	0·63, 2·07	93	1·17	0·73, 1·89	1·11	0·65, 1·92	1·10	0·70, 1·72	1·01	0·61, 1·67
* P*_trend_^‡^			0·78		0·72			0·52		0·69		0·67		0·96	
Grains and dairy products															
T1	53	76	1·00 (ref)		1·00 (ref)		96	1·00 (ref)		1·00 (ref)		1·00 (ref)		1·00 (ref)	
T2	64	84	1·06	0·64, 1·76	1·16	0·64, 1·95	78	0·67	0·42, 1·08	0·64	0·38, 1·08	0·67	0·43, 1·05	0·65	0·41, 1·04
T3	63	79	1·22	0·73, 2·07	1·49	0·82, 2·72	83	0·76	0·48, 1·24	0·80	0·46, 1·39	0·68	0·43, 1·07	0·60	0·36, 0·98
* P*_trend_^‡^			0·44		0·19			0·28		0·42		0·09		0·04	
Processed meat and discretionary foods															
T1	71	67	1·00 (ref)		1·00 (ref)		87	1·00 (ref)		1·00 (ref)		1·00 (ref)		1·00 (ref)	
T2	62	87	1·32	0·80, 2·17	1·64	0·93, 2·89	77	1·06	0·66, 1·69	1·03	0·60, 1·75	0·76	0·48, 1·20	0·82	0·50, 1·34
T3	47	85	2·02	1·18, 3·46	2·60	1·32, 5·09	93	1·88	1·13, 3·14	2·13	1·13, 4·00	0·97	0·60, 1·55	0·98	0·56, 1·73
* P*_trend_^‡^			0·01		0·005			0·02		0·03		0·93		0·96	

T1, tertile 1; T2, tertile 2; T3, tertile 3.

* OR and 95 % CI from multinomial logistic regression analysis, adjusted for age and sex.

† OR and 95 % CI from multinomial logistic regression analysis, adjusted for age, sex, total energy intake, BMI category, smoking status, personal polyp history, family history of bowel cancer, previous colonoscopy, current alcohol consumption and regular aspirin use.

‡ *P*-value for linear trend obtained by treating the factor category variable as continuous.

To assess whether any particular food groups accounted for the associations observed, we adjusted the model for the ‘Processed meat and discretionary food’ pattern for the food groups that contributed substantially to the pattern (i.e. a factor loading > 0·3). Among CA cases (Table 5), further adjustment for ‘savoury snacks and sauces’ attenuated the OR estimates for the highest intake group to 1·77 (95 % CI 0·82, 3·86), and the trend was no longer statistically significant. Among SSL cases (Table 6), further adjustment for ‘savoury snacks and sauces’ and ‘high-energy drinks’ attenuated the OR estimates for the highest intake group to 1·76 (95 % CI 0·86, 3·60) and 1·82 (95 % CI 0·90, 3·68), respectively, and the trends were no longer statistically significant. Adjusting for the other food groups did not substantially change the estimates for either CA or SSL cases. Therefore, the association between the ‘Processed meat and discretionary food’ pattern and polyp risk is at least partly due to the intake of savoury snacks and sauces (CA and SSL) and high-energy drinks (SSL only).

**Table 5.** OR and 95 % CI for the association between the processed meat and discretionary foods dietary pattern and conventional adenoma (CA) risk in 239 CA cases and 180 polyp-free controls, adjusted for food group items with a high factor loading (> 0·3)

		Tertile group 2		Tertile group 3		
Dietary patterns modelled with food groups added to the model	Tertile group 1 (Reference group)	OR	95 % CI	OR	95 % CI	*P*_for trend_^†^
Without additional adjustment for food groups	1·00	1·64	0·93, 2·89	2·60	1·32, 5·09	0·005
Processed meat and discretionary foods^*^	1·00	1·70	0·96, 3·01	2·64	1·34, 5·20	0·005
High-fat dairy products	1·00	1·72	0·96, 3·07	2·62	1·32, 5·21	0·006
Potato	1·00	1·72	0·97, 3·06	2·68	1·35, 5·32	0·005
Red meat	1·00	1·77	0·99, 3·17	2·77	1·37, 5·63	0·005
Processed meat	1·00	2·03	1·11, 3·71	3·84	1·76, 8·38	0·0007
Refined grains	1·00	1·71	0·97, 3·03	2·68	1·35, 5·33	0·005
Sweet snacks and spreads	1·00	1·85	1·03, 3·34	3·16	1·53, 6·51	0·002
Savoury snacks and sauces	1·00	1·31	0·71, 2·44	1·77	0·82, 3·86	0·15
Fat spreads	1·00	1·71	0·96, 3·06	2·68	1·32, 5·48	0·007
Discretionary fat	1·00	1·81	1·01, 3·25	2·71	1·32, 5·54	0·006
Alcoholic beverages	1·00	1·66	0·94, 2·95	2·46	1·24, 4·90	0·01
High-energy drinks	1·00	1·98	1·08, 3·65	3·37	1·57, 7·20	0·002

* OR and 95 % CI from multinomial logistic regression analysis, adjusted for age, sex, total energy intake, BMI category, smoking status, personal polyp history, family history of bowel cancer, previous colonoscopy, current alcohol consumption and regular aspirin use.

† *P*-value for linear trend obtained by treating the factor category variable as continuous.

**Table 6.** OR and 95 % CI for the association between the processed meat and discretionary foods dietary pattern and sessile serrated lesion (SSL) risk in 257 sessile serrated adenoma cases and 180 polyp-free controls, adjusted for food group items with a high factor loading (> 0·3)

		Tertile group 2		Tertile group 3		
Dietary patterns and food groups included in the model	Tertile group 1 (Reference group)	OR	95 % CI	OR	95 % CI	*P*_for trend_^†^
Without additional adjustment for food groups	1·00	1·03	0·60, 1·75	2·13	1·13, 4·00	0·03
Processed meat and discretionary foods^*^	1·00	1·03	0·60, 1·75	2·13	1·13, 4·00	0·02
High-fat dairy products	1·00	1·02	0·60, 1·75	2·16	1·13, 4·12	0·02
Potato	1·00	1·05	0·61, 1·80	2·16	1·14, 4·09	0·02
Red meat	1·00	0·96	0·55, 1·65	1·96	1·02, 3·74	0·05
Processed meat	1·00	1·17	0·66, 2·08	2·70	1·32, 5·52	0·007
Refined grains	1·00	1·06	0·61, 1·82	2·55	1·33, 4·90	0·007
Sweet snacks and spreads	1·00	1·07	0·62, 1·84	2·27	1·17, 4·39	0·03
Savoury snacks and sauces	1·00	0·86	0·48, 1·54	1·76	0·86, 3·60	0·13
Fat spreads	1·00	1·03	0·60, 1·79	2·16	1·09, 4·26	0·03
Discretionary fat	1·00	1·06	0·61, 1·82	2·06	1·06, 3·97	0·04
Alcoholic beverages	1·00	1·02	0·60, 1·75	2·04	1·07, 3·86	0·04
High-energy drinks	1·00	0·95	0·54, 1·66	1·82	0·90, 3·68	0·11

* OR and 95 % CI from multinomial logistic regression analysis, adjusted for age, sex, total energy intake, BMI category, smoking status, personal polyp history, family history of bowel cancer, previous colonoscopy, current alcohol consumption and regular aspirin use.

† *P*-value for linear trend obtained by treating the factor category variable as continuous.

## Discussion

In this case–control study, we investigated lifestyle factors and dietary patterns in relation to the risk of SSL and CA in an Australian population. Considering that up to two-thirds of colorectal cancers may be attributable to dietary and lifestyle risk factors^(8)^, this investigation contributes important insights for reducing the risk of developing polyps with malignant potential.

In our study, being a current smoker was associated with an increased risk of SSL, while regular use of anti-inflammatory medications was associated with reduced risk of SSL. Previous observational studies also report an increased risk of SSL with smoking and a reduced risk with anti-inflammatory medications, supporting the external validity of our findings^(8,12,13)^. Unlike our study, regular use of aspirin has been associated with a reduced risk of serrated polyps in general^(33)^. In research using a *BRAF* mutant, serrated pathway mouse model, aspirin did not reduce the incidence of murine serrated lesions^(34)^. Although aspirin use may not be relevant for chemoprevention in the serrated pathway, it did not increase the risk of cancer and has been shown to reduce the risk of CA^(35,36)^. While not investigated as a risk factor in our study, we identified that SSL cases were more likely to be younger than CA cases. This is consistent with previous work suggesting that SSL can develop at a young age^(37)^. Other research has reported an increased risk of SSL with higher intakes of red meat and well-done red meat^(14)^; however, these associations were not observed in our study.

Compared with CA, use of HRT, current alcohol consumption and being overweight were protective factors for SSL, and being taller was associated with increased SSL risk. Previous research has reported a protective effect for long-term use of HRT and therapies with progesterone on the risk of developing high-risk serrated polyps but not high-risk adenomatous polyps^(38)^. While current alcohol use is an established risk factor for CA^(39)^, which we also observed in our study, the evidence to date indicates that alcohol intake may not be a particular risk factor for SSL. A meta-analysis in 2017 suggested that alcohol intake was a risk factor for SSL^(8)^, but the analysis was based on only three older studies, whereas more recent studies have indicated no association between alcohol consumption and SSL risk^(11,13)^. In a recent study from China, being overweight was associated with serrated and CA, with a higher risk estimate for serrated adenoma^(40)^. Regarding taller height, previous evidence has shown that persons of Anglo-Celtic origin are more likely to be affected by SSL than people of southern European origin^(41)^ (i.e. being taller may be a surrogate for Northern European ethnicity).

Risk factors for CA are relatively well established, including being male, older age, African American ethnicity, alcohol consumption, smoking and obesity^(39,42)^. In our study, participants were predominantly of Caucasian ethnicity, and CA cases were more likely to be male, older, currently consuming alcohol, former smokers and overweight or obese compared with polyp-free controls. However, after adjusting for confounders, only current alcohol consumption was associated with CA risk. It is not clear why smoking and obesity were not confirmed as risk factors in our data.

To our knowledge, this is the first study to investigate dietary patterns in relation to histologically confirmed SSL. We found that a dietary pattern featuring processed meats, ready-made convenience foods and high-energy drinks was associated with an increased risk of SSL and CA. The difference between the effect estimates for SSL and CA risk was not significant, suggesting dietary components of the ‘Processed meat and discretionary food’ pattern are relevant to both the serrated and adenoma-carcinoma pathways. This finding is supported by a meta-analysis of seven observational studies in which unhealthy dietary patterns were associated with a 24 % higher risk of developing any type of colorectal adenoma^(19)^. The unhealthy dietary patterns in this meta-analysis were identified in studies among American, African American, European and Asian populations and featured processed meats, refined grains and ready-made convenience foods^(19)^. A diet that is more pro-inflammatory (a likely characteristic also of a processed meat and discretionary food dietary pattern) has also been associated with increased risk of SSL and CA^(43)^. This suggests that the increased risk of colorectal adenoma with adherence to an unhealthy dietary pattern is reproducible, despite differences in population characteristics and the influence of contextual factors, such as geographical location and culture, on food supply and intake.

Several mechanisms have been proposed for the increased risk of colorectal adenoma with higher adherence to unhealthy dietary patterns. Preservatives in processed meats, including sulphur, nitrites and nitrates, may have a mutagenic effect on colorectal epithelial cells, promoting abnormal tissue growth^(44)^. In a large prospective study in North America, a ‘Western’ dietary pattern featuring processed meats was more strongly associated with microsatellite stable and CpG Island Methylator Phenotype (CIMP)-low or negative colorectal tumours, characteristics of the CA-carcinoma pathway^(9,45)^. These findings are reflected in our study, which showed the ‘Processed meats and discretionary foods’ pattern was more strongly associated with CA; however, the greater risk estimate for CA was not significant in case-case comparisons. Other key food groups in the ‘Processed meats and discretionary foods’ pattern included refined grains, ready-made convenience foods, fat spreads, discretionary fats and high-sugar foods and beverages. These food groups suggest a higher intake of saturated fats and refined carbohydrates, which have been associated with hyperinsulinemia^(46)^. If prolonged, hyperinsulinemia can induce insulin resistance and elevate levels of insulin-like growth factor-1, which may promote cell proliferation and inhibit apoptosis, especially in the colon^(44,47)^.

Compared with CA, a dietary pattern characterised by wholegrains, low-fat dairy products, nuts, seeds and oily fish may relatively protect against the development of SSL. These findings contribute important food-based evidence as previous studies have focused on single nutrients with mixed findings. For example, considering the food group composition of the ‘Grains and dairy products’ pattern identified in this study, it is likely that this pattern is associated with higher intakes of folate, calcium, fibre and *n*-3 fatty acids. In an analysis of dietary factors in relation to SSL specifically, higher intakes of fibre and folate were associated with a reduced risk of SSL, but these associations did not remain when other risk factors were included in the model^(11)^. Marine *n*-3 fatty acids have been associated with a lower risk of serrated polyps in general^(48)^, although these findings were not replicated in a randomised controlled trial^(49)^. Thus, our study provides preliminary insights into dietary factors associated with SSL development and accounts for the synergistic effects of nutrients and foods in health and disease outcomes.

Overall, our findings support the use of a total diet approach in the prevention of colorectal cancer and provide insights into the interplay of dietary factors in the development of colorectal cancer precursors. This study builds on previous studies that have investigated individual foods and nutrients and provides important evidence for informing food-based dietary guidelines. Our findings regarding diet and lifestyle factors associated with risk of SSL have important clinical and public health relevance as SSL have a very flat morphology and, compared with CA, contribute to a greater proportion of interval colorectal cancers that arise between colonoscopies^(7)^.

### Strengths and limitations

Strengths of this study include the relatively large number of SSL cases and the synchronous prospective recruitment of CA cases and polyp-free controls from the same hospital schedules. Another important strength is the ascertainment of cases and controls; members of the research team, and other clinicians working at the collaborating hospital sites, have well-established, long-term expertise in detection, resection and histological assessment of serrated lesions, achieving high SSL detection rates unlike many older studies^(4)^. Further, the control group included people with a colonoscopically confirmed absence of polyps. This is expected to have minimised potential misclassification of cases and polyp-free controls. While the study participant rate was lower in the polyp-free control group, it was relatively similar to cases. Therefore, it is unlikely that different levels of participation explain the differences between cases and controls. Our findings are similar to studies in other population groups that have investigated lifestyle and diet-related factors in relation to the risk of colorectal adenoma or cancer, supporting the external validity of our findings. Future research should consider vaping and the use of e-cigarettes in relation to SSL risk, a lifestyle factor that was not common at the time of data collection in the current or previous studies.

A limitation of this study, and of most previous epidemiological studies of SSL risk factors, is that there was no assessment of the exact timing of exposure to medication and lifestyle factors in relation to the trajectory of development from precursor lesion to detectable serrated lesion. However, that investigation would require frequent, repeated assessments in a longitudinal study design, which is difficult to achieve in human studies given the invasive nature of colonoscopy. Although validated instruments were used, case–control studies rely on a person’s recall, which can be subject to bias^(50)^. There was, however, a high correlation between self-reported use of multivitamin supplements and red-cell folate and vitamin B_12_ status in our study participants (details not shown). It is also possible that the polyp-free controls may not be representative of the general population, as they were selected from people who underwent a colonoscopy for clinical reasons. However, colonoscopy screening for research, without medical indication, is not feasible because it is too invasive and costly. Participation was relatively lower in the polyp-free control group, which may have influenced our ability to detect weaker associations that may exist. However, this is one of the largest studies to date of well-defined SSL cases, and important associations were detected despite the lower number of polyp-free participants.

Our use of dietary patterns to measure dietary exposure captures the synergistic interactions between dietary factors that potentially confound risk estimates for single nutrients and foods^(18)^. To reduce subjectivity involved in deriving *a posteriori* dietary patterns, groupings of FFQ items were guided by published literature and the Australian Dietary Guidelines^(51)^. We used established criteria and best practice to determine the number of dietary patterns to retain and their interpretation. While the proportion of total variance explained by our dietary patterns (28 %) was slightly higher than the 20–25 % in previous studies of colorectal adenoma risk, it indicates that there are other dietary patterns in the population that may also influence adenoma risk^(15)^. Further, previous studies have identified differential risk associations by sex^(19)^; however, due to our sample size, we were unable to perform such subgroup analyses. Additionally, dietary intake in the past year may not represent long-term habitual diet, and timing of dietary exposure may also influence carcinogenesis^(44,52)^. For example, the mutagenic effect of red meat may be more potent in the initial stages of adenoma development than in the latter stages of neoplastic growth^(44)^. However, such temporal associations cannot be assessed in case–control studies.

### Conclusions

This study identified that regular use of anti-inflammatory medications is associated with reduced risk of SSL, while being a current smoker is associated with increased risk of SSL. Compared with CA, use of HRT, current alcohol consumption and being overweight may be associated with lower SSL risk and being taller with increased SSL risk. A dietary pattern characterised by the consumption of processed meats, ready-made convenience foods and high-energy drinks may increase the risk of developing SSL and CA. Compared with CA, a dietary pattern featuring wholegrains, low-fat dairy products, nuts, seeds and oily fish is associated with a reduced risk of developing SSL, the polyp type that is more likely to be missed during colonoscopy due to its flat morphology. This study is the first to report on dietary patterns in relation to SSL risk, contributing to the evidence base for a total diet approach to prevention of colorectal cancer, beyond a focus on single nutrients and foods. Evidence of SSL risk factors from this and other epidemiological studies can be combined with clinical and molecular data to individualise therapeutic and preventive strategies. Further research using histologically well-defined SSL could be conducted to investigate the longer-term effects of dietary patterns and lifestyle factors, including vaping and use of e-cigarettes, on the risk of developing SSL and colorectal adenomas, as well as elucidating differences by sex.

## Supporting information

van der Pols et al. supplementary material van der Pols et al. supplementary material

## Supplementary material

For supplementary material/s referred to in this article, please visit https://doi.org/10.1017/S0007114525103747.
