# Supplementary material for: Dietary pattern and lifestyle risk factors for sessile serrated precursors to colorectal cancer
Source: Br J Nutr. 2025 Jun 24;134(1):35–46. doi: 10.1017/S0007114525103747 (PMC12379116; doi:10.1017/S0007114525103747)
Supplement: van der Pols et al. supplementary material [file S0007114525103747sup001.docx]

**Supplementary Table 1.** Food groups used in dietary pattern analyses.

| **Food group** | **Food items** |
| --- | --- |
| Low fat dairy | Skim milk, low-fat milk, low-fat yoghurt, low-fat cheese (e.g., cottage, ricotta) |
| High fat dairy | Whole milk, regular yoghurt, cream, ice cream, custard, regular or creamy cheese (e.g., cheddar, brie) |
| Green leafy vegetables | Spinach, silverbeet, lettuce |
| Cruciferous vegetables | Cabbage, cauliflower, broccoli, Brussel sprouts, coleslaw |
| Orange vegetables | Carrot, pumpkin, sweet potato |
| Fruiting vegetables | Tomato, eggplant, zucchini, squash, avocado |
| Other vegetables | Mushrooms, celery, bean sprouts, green beans, peas, corn |
| Potato | White potato (boiled, baked, roasted, or mashed) |
| Legumes | Baked beans**,** other beans, or lentils |
| Pickles | Olives, gherkins, pickled vegetables |
| High vitamin A & C fruit | Orange, mandarin, grapefruit, mango, pawpaw, strawberries, other berries, melon, pineapple, apricots |
| Other fruit | Apple, pear, banana, grapes, peaches, plums, nectarines, sultanas, dates, prunes and other fresh, frozen, canned, or dried fruit |
| Juice | Orange juice, apple juice, other fruit juice, tomato juice |
| Red meat | Beef, lamb or pork as main dish or mixed dish, minced meat, hamburger patty, liver |
| Processed meat | Ham, salami, sausages, bacon, Frankfurt |
| Poultry | Chicken with or without skin |
| Oily fish | Tuna, sardines, salmon, or other dark meat fish (canned or fresh) |
| Other fish & seafood | White fish, prawns, crabs, scallops, other fish, and seafood |
| Eggs | Eggs (boiled, scrambled, fried) |
| Nuts & seeds | Nuts, seeds (e.g., pumpkin, sesame) |
| Soy products & meat alternatives | Soybean, soymilk (regular, low-fat), soy-based meat substitutes (e.g., tofu, tempeh, miso), nut-based meat substitutes |
| Wholegrains | Wholemeal, mixed grain or soy and linseed bread or toast, porridge, brown rice |
| Refined grains | White bread or toast, scones, pikelets, white rice, pasta, noodles, crispbread, crackers, tortillas, pita bread, other refined grains (e.g., couscous), cold breakfast cereal |
| Sweet snacks & spreads | Bakery items (cake, tart, pie, pastry, pavlova, cheesecake, sweet roll/bun), biscuits (plain, flavoured, coated), cereal/muesli bar, chocolate, lollies, peanut paste, jam, marmalade, syrups, honey, sugar |
| Savoury snacks & sauces | Pizza, sausage roll, meat pie, hot chips, wedges, hash browns, potato chips, corn chips, twisties, fish cakes or fish sticks, other salad dressings, tomato sauce/ketchup, pasta sauces (tomato, cream-based) |
| Fat spreads | Butter, margarine, mayonnaise |
| Discretionary fats | Visible fat on meat, food fried at home, fried take-out foods, ready-made foods |
| Vegemite | Vegemite, Marmite, Promite |
| Alcoholic beverages | Red wine, white wine, beer, spirits, liqueurs, and other alcoholic beverages |
| High-energy drinks | Cola drinks, lemonade, other sugar-sweetened soft drinks, cordial, sports drinks, energy drinks |
| Low-energy drinks | Diet cola drink, other diet soft drinks |
| Coffee | Coffee (regular, decaffeinated) |
| Tea | Tea (black, green, herbal) |
| Water | Water |

**Supplementary Table 2.** Factor loading matrix of food groups for the three dietary patterns identified^a^

| **Food group** | **Vegetables and protein sources** | **Grains and dairy** | **Processed meat and discretionary foods** |
| --- | --- | --- | --- |
| Low fat dairy |  | **0.47** |  |
| High fat dairy |  |  | **0.36** |
| Green leafy vegetables | **0.45** | **0.48** |  |
| Cruciferous vegetables | **0.70** |  |  |
| Orange vegetables | **0.73** |  |  |
| Fruiting vegetables | **0.53** | **0.36** |  |
| Other vegetables | **0.74** |  |  |
| Potato | **0.51** | -0.21 | **0.36** |
| Legumes |  | **0.43** |  |
| Pickles |  | **0.41** | 0.20 |
| High vitamin A & C fruit | **0.48** | **0.38** |  |
| Other fruit | **0.37** | **0.40** |  |
| Juice | 0.21 |  | 0.26 |
| Red meat | **0.43** | -0.28 | **0.45** |
| Processed meat |  | -0.22 | **0.62** |
| Poultry | **0.38** |  | 0.28 |
| Oily fish | 0.26 | **0.47** |  |
| Other fish & seafood | **0.44** |  |  |
| Eggs | 0.21 | 0.22 |  |
| Nuts & seeds |  | **0.54** |  |
| Soy products & meat alternatives |  | **0.38** | -0.23 |
| Wholegrains |  | **0.58** |  |
| Refined grains |  | **0.39** | **0.35** |
| Sweet snacks & spreads |  |  | **0.45** |
| Savoury snacks & sauces |  |  | **0.67** |
| Fat spreads |  |  | **0.51** |
| Discretionary fats |  | -0.21 | **0.40** |
| Vegemite |  |  | 0.25 |
| Alcoholic beverages |  |  | **0.34** |
| High-energy drinks |  |  | **0.55** |
| Low-energy drinks |  |  |  |
| Coffee |  | 0.26 |  |
| Tea | 0.21 | **0.35** |  |
| Water |  | 0.26 |  |

^a^Absolute factor loadings <0.20 are excluded for simplicity. Bold values indicate foods with
the highest contribution to each pattern (absolute factor loading ≥0.3).

.
